# Japanese encephalitis in Nepal: closing the adult immunity gap

**DOI:** 10.1186/s41182-026-01011-8

**Published:** 2026-07-06

**Authors:** Alisha GC, Arzu Kunwar, Ankit Jaiswal, Binod Rayamajhee, Binod GC

**Affiliations:** 1 Nepalgunj Medical College, Banke, Nepal; 2 Department of Neuroscience, UConn Health, Farmington, CT USA; 3 School of Veterinary Medicine, University of Pennsylvania, Philadelphia, PA USA; 4 Faculty of Medicine, Macquarie University, Sydney, Australia; 5 Department of Infection and Immunology, Kathmandu Research Institute for Biological Sciences, Lalitpur, Nepal; 6 Department of Biomedical Sciences, Cedars-Sinai Medical Center, Los Angeles, CA USA

## Abstract

Japanese encephalitis (JE) in Nepal is undergoing an epidemiological shift from a predominantly pediatric, Terai-centered disease to a geographically broader threat affecting susceptible adults. Despite successful childhood vaccination, the 2025 resurgence, with 175 confirmed cases and 34 deaths, exposed a major immunity gap among older cohorts, particularly adults over 40 years. Expanding transmission into hill districts and higher-altitude areas, likely influenced by climate variability, land-use change, and vector ecology, further challenges existing control strategies. Because JE overlaps clinically and radiologically with other endemic encephalitides, rapid laboratory confirmation is essential. Nepal’s emerging adult vaccination policy should be integrated with climate-informed surveillance, One Health data systems, vector control, and survivor rehabilitation before future monsoon-driven outbreaks.

Japanese encephalitis (JE) remains an important but changing public health threat in Nepal. More than 3 billion people in Asia and the Western Pacific live in areas at risk of infection with Japanese encephalitis virus (JEV), a mosquito-borne flavivirus maintained in an enzootic cycle involving *Culex* mosquitoes, pigs, and wading birds [1]. Although most infections are asymptomatic, clinical diseases are severe: case fatality can approach 30%, and neurological or psychiatric sequelae affect many survivors [1]. Historically, JE has been regarded as a childhood disease, but recent events in Nepal suggest that this framing is now incomplete [2, 3].

Nepal has reported JE since 1978, with the heaviest burden traditionally concentrated in the Terai, especially districts bordering India [4]. Irrigated agriculture, monsoon flooding, pig rearing, expanding settlement in low-altitude plains, and limited access to timely diagnostics have supported recurrent outbreaks [4]. Hospital-based acute encephalitis syndrome (AES) surveillance has helped define this burden, but it almost certainly underestimates transmission because mild, subclinical, and non-hospitalized infections are missing. Spatial analyses have shown that JE and AES risk in Nepal are dynamic rather than fixed, and recent estimates from Bangladesh, including substantial numbers of inapparent infections, reinforce the need to look beyond notified clinical cases when assessing regional risk [4, 5].

The 2025 resurgence in Nepal is particularly concerning because it points to both an epidemiological and demographic transition. Nepal reported 175 confirmed cases and 34 deaths in 2025, compared with 80 cases and 23 deaths in 2024 [3]. Approximately 76% of reported JE deaths in late 2025 occurred in people older than 40 years [3]. This pattern should not be interpreted as simple vaccine failure. A more biologically plausible interpretation is a cohort effect created by age-targeted vaccination and changing force of infection: children and younger adults were more likely to benefit from campaign vaccination, routine childhood immunization, and/or immune boosting through prior subclinical exposure, whereas many adults now older than 40 years may have aged outside the original vaccine-eligible cohorts or lived in settings where vaccination was not uniformly implemented [6, 11]. Nepal’s SA 14-14-2 program substantially reduced disease, with post-campaign JE incidence 78% lower than expected through 2014, but protection was concentrated in vaccinated districts and eligible age groups rather than distributed evenly across the adult population [11]. Thus, the lower recent mortality among those younger than 40 years is most consistent with heterogeneous cohort immunity, not with absence of exposure or universal protection.

The possible contribution of waning vaccine-induced immunity should be interpreted cautiously. In JE vaccine studies, a 50% plaque-reduction neutralization test titer of at least 1:10 is commonly used as a serological correlate of protection [14]. Field effectiveness data from Nepal support durable protection after SA 14-14-2 vaccination: a single dose was 99.3% effective shortly after vaccination and approximately 96% effective five years after vaccination in Nepalese children [12, 13]. However, antibody persistence and clinical protection are not identical. In Bangladesh, only 9.3% and 12.4% of children had measurable seroprotective neutralizing antibody three and four years after a primary CD-JEV dose, but 91.4% and 98.1% were seroprotected 7 and 28 days after a booster, indicating immune memory despite low circulating antibody in many vaccinees [14]. A systematic review similarly found persistence of seropositivity after primary vaccination but strong boostability after booster doses [15]. Therefore, waning antibody could contribute to susceptibility in some individuals, particularly where natural boosting is limited, but Nepal's recent adult mortality pattern cannot be attributed to waning immunity from aggregate case data alone. Disentangling waning immunity from historical non-vaccination requires age-stratified vaccination histories, serosurveys, and investigation of laboratory-confirmed breakthrough JE among documented vaccinees.

The geography of JE risk in Nepal is also changing. The disease is no longer confined to the Terai: cases have been reported from the Kathmandu valley, hill districts, and higher-altitude areas [4, 7, 16]. Re-emergence is therefore unlikely to be explained by immunity gaps alone. Climate variability, rising temperatures, altered rainfall, irrigated agriculture, peri-urban settlement, pig-rearing practices, and changing human mobility may be reshaping the ecological interface among Culex vectors, amplifying hosts, and humans [4, 7, 17]. Evidence of JEV circulation in Culex tritaeniorhynchus and pigs in high-altitude regions of Tibet supports the biological plausibility of transmission in areas previously considered too cool for sustained risk [8]. In Nepal, the greatest recent burden has been reported in Lumbini, followed by Gandaki, Bagmati, and Koshi provinces, with transmission peaking during the June–October monsoon, especially in August [3, 7].

Clinical management remains difficult. JE can present seizures, altered consciousness, cranial nerve involvement, parkinsonism, and acute flaccid paralysis, and severe cases often require intensive respiratory and neurological support [1]. No specific antiviral treatment is available. The clinical syndrome overlaps with other endemic infections, including scrub typhus, tuberculous meningitis, dengue-associated encephalopathy, and other viral encephalitides [1, 9]. Diagnostic uncertainty is compounded by radiological overlap: bilateral thalamic lesions, often considered suggestive of JE, have recently been described in scrub typhus meningoencephalitis in Nepal [9]. This overlap makes syndromic diagnosis inadequate. Strengthening laboratory confirmation through CSF and serum IgM testing, referral testing where needed, and integrated testing algorithms for AES should be treated as core outbreak-control infrastructure, not as optional tertiary-care capacity [1, 9].

Nepal's 2026 response marks an important policy shift. Targeted vaccination of adults aged 35 years and older in high-risk districts, including Nawalparasi East, acknowledges the adult immunity gap and should be expanded using risk stratification rather than administrative convenience alone [10]. Districts should be prioritized according to recent AES/JE incidence, vector density, pig and water-bird ecology, climate suitability, health-care access, and age-stratified vaccination history [4, 7, 10, 11]. Extension of JE vaccination to all age groups in all endemic areas should not be assumed to be optimal without local evidence. A more defensible near-term approach is targeted adult catch-up vaccination for historically unvaccinated cohorts in districts with recent transmission, followed by expansion if age-specific incidence, seroprevalence, and cost-effectiveness data support broader eligibility [5, 11, 14, 15]. Periodic antibody testing of previously vaccinated individuals is unlikely to be feasible as a routine population-level intervention because neutralization testing is technically demanding and protection may persist through immune memory even when circulating antibody is low [14, 15]. Instead, sentinel age-stratified serosurveillance, linkage of cases to vaccination histories, and systematic investigation of suspected vaccine failures should guide future booster policy.

Nepal now needs a JE strategy designed for a changing climate and an ageing susceptible population. The priorities are clear: catch-up vaccination for unprotected adults in high-risk districts; climate-informed AES surveillance; age-stratified serosurveillance and vaccination-history linkage; linked human, animal, and entomological data systems; rapid laboratory confirmation; vector control around irrigated and peri-urban settlements; and rehabilitation services for survivors. The 2025–2026 resurgence should be read as a warning that childhood immunization alone is no longer sufficient [3, 10]. In Nepal, JE control will depend on closing the adult immunity gap before the next monsoon-driven outbreak exposes it again.

## Data Availability

No datasets were generated or analyzed during the current study.
